# Cytochrome P450 1B1 Overexpression in Cervical Cancers: Cross-sectional Study

**DOI:** 10.2196/31150

**Published:** 2021-10-12

**Authors:** Fatemah O F O Alshammari, Yousef M Al-saraireh, Ahmed M M Youssef, Yahya M Al-Sarayra, Hamzeh Mohammad Alrawashdeh

**Affiliations:** 1 Department of Medical Laboratory Technology Faculty of Health Sciences The Public Authority for Applied Education and Training Shuwaikh Kuwait; 2 Department of Pharmacology Faculty of Medicine Mutah University Al-Karak Jordan; 3 Department of Pharmacology Faculty of Pharmacy Mutah University Al-Karak Jordan; 4 Al-Karak Governmental Hospital Jordan Ministry of Health Al-Karak Jordan; 5 Department of Ophthalmology Sharif Eye Centers Irbid Jordan

**Keywords:** cancer, cervical cancer, cytochrome P450, cytochrome 1B1, immunohistochemistry, toxicity, therapies, molecular, tumor, cytochrome, cervix

## Abstract

**Background:**

Current standard treatments for patients with recurrent cervical cancer are not very effective and are associated with severe toxicity. Recently, the rational approach for the discovery of new therapies for cervical cancer is based on the alterations in the molecular biology of cancer cells. One of the emerging molecular changes in cancer cells is the aberrant expression of cytochrome P450 1B1 (CYP1B1). This unique enzyme has been reported to be selectively overexpressed in several cancers.

**Objective:**

The aim of this study was to examine CYP1B1 expression in cervical cancers and to assess the enzyme’s relationship with several clinicopathological features.

**Methods:**

Immunohistochemistry was performed to examine CYP1B1 expression in 100 patient samples with cervical cancer and 10 patient samples with normal healthy cervical tissues.

**Results:**

CYP1B1 was expressed in the majority of the cervical cancer samples (91/100, 91.0%) but not in normal healthy cervical samples. The difference in the expression of CYP1B1 between healthy and tumorous cervical tissues was significant (*P*=.01). Moreover, the frequency of CYP1B1 expression was found to be significantly higher in patients with advanced grades of the disease (*P*=.03) and in patients having metastasis to the lymph nodes (*P*=.01). Surprisingly, there was a significantly higher expression of CYP1B1 in patients with a high prevalence of human papilloma virus 16/18 (*P*=.04).

**Conclusions:**

The differential profile of CYP1B1 expression between cervical cancer tissues and normal cervical tissues suggests that CYP1B1 may be used as a target for future therapeutic exploitations.

## Introduction

Cervical cancer is the fourth most frequent cause of cancer deaths and morbidity in women around the world [1,2]. According to the GLOBOCAN 2018 study by the International Agency for Research on Cancer, cervical cancer prevalence surpasses 570,000 new cases and 311,000 deaths worldwide [3]. The primary cause of cervical cancer development is persistent infection with human papilloma virus (HPV), in particular, HPV 16 and HPV 18, although this is not the only factor. These 2 aggressive types of viruses are responsible for causing about 70% of cervical cancers [4,5]. In the past decades, the incidence of HPV in cervical cancer has decreased owing to proper cervical screening and implementation of HPV vaccination programs. Nevertheless, the incidence of cervical cancer keeps rising in low-income countries because of the lack or improper implementation of cervical screening and vaccination programs against HPV [2,5]. Thus, new targeted therapies are urgently needed for better management of cervical cancer.

Several metabolic enzymes, particularly members of the cytochrome P450 (CYP) superfamily, are being investigated for their potential role in tumor malignancy [6-8]. Of recent interest is a novel member of the *CYP1* gene family, CYP1B1, which is an extrahepatic enzyme that is engaged in the metabolism of several endogenous and xenobiotic compounds [9]. CYP1B1 has been implicated in the oxidation of steroids, particularly estrogen and melatonin. Moreover, many xenobiotics such as aromatic amines and polycyclic aromatic hydrocarbons are oxidized by CYP1B1 to active carcinogenic products [10]. Importantly, CYP1B1 has recently been proposed as a universal tumor antigen owing to its selective overexpression in tumors compared to negative or undetectable levels of protein expression in the corresponding normal tissues [11]. A high frequency of CYP1B1 expression has been reported in cancers of the brain, breast, prostate, colon, bladder, muscle (rhabdomyosarcoma), ovary, and lungs (non–small cell lung cancer) [11-22]. Moreover, metastatic tumors show an elevated level of CYP1B1 expression [14]. This expression is highly prevalent in advanced grades and late stages of the disease [23]. Although CYP1B1 mRNA is found in many healthy tissues, its related protein expression is rarely identified [11,24]. Additionally, in vitro and in vivo studies using tumor cell line models have shown that CYP1B1 overexpression promotes cell invasion, migration, and proliferation, and hampers cell death. Moreover, overexpression of CYP1B1 is associated with larger tumor sizes, recurrent lymph node metastasis, advanced tumor grades, and lymphovascular invasion [25-27]. Intriguingly, overexpression of CYP1B1 was found to decrease the sensitivity of tumor cells to anticancer drugs such as paclitaxel and docetaxel, resulting in drug resistance [28,29]. Thus, the differential and tumor-specific expression of CYP1B1 provides a novel potential for the development of new anticancer therapeutics.

Several studies have comprehensively and quantitatively analyzed the role of CYP1B1 polymorphisms in the increased risk and development of cancer [30]. G119T and A453G polymorphisms have been reported to be associated with an increased risk of lung, prostate, colorectal, breast, endometrial, and bladder cancers [27]. Moreover, Asn^453^Ser, Arg^48^Gly, Val^432^Leu, and Ala^119^Ser have been shown to be related to increased estrogen metabolism, resulting in genotoxic metabolites that lead to hormone-induced tumors [15]. For instance, polymorphisms of Asn^453^Ser and Arg^48^Gly were linked with endometrial cancer risk and Ala^119^Ser with breast cancer risk. The polymorphism Leu^432^Val was linked to an increased risk of ovarian, breast, and endometrial cancers [31]. Conversely, another study showed no link between CYP1B1 polymorphisms Arg^48^Gly, Ala^119^Ser, and Asn^453^Ser and the risk of breast cancer [32]. These CYP1B1 polymorphisms are believed to increase cancer risk through molecular mechanisms such as enhanced progesterone and estrogen receptor signaling, which is also known to affect chemotherapy response [15]. This particularly applies to some CYP1B1 polymorphisms causing a weak response to anthracycline agents, while low polymorphisms were found to improve response to chemotherapeutic agents [33].

Given that no previous studies have examined the expression of CYP1B1 in cervical cancers, this study aims to assess the aberrant expression of CYP1B1 in cervical cancer and its relation to baseline demographic and clinicopathologic features.

## Methods

### Tissue Specimens

A total of 110 formalin-fixed and wax-embedded tissue specimens of human cervical cancer and normal cervix were collected from the pathology department of the King Hussein Medical Hospital, Royal Medical Services, Amman and the King Abdullah University Hospital, Irbid, Jordan. None of the patients included in this study had received chemotherapy or radiotherapy. Clinical tissue samples comprised 95 squamous cell carcinomas, 3 adenocarcinomas, 2 endometrioid adenocarcinomas, and 10 tissues of normal cervices. All relevant patient information on age, tumor histopathology, tumor grade, clinical stage, and expression of HPV 16/18 and Ki67 were obtained from patient reports. The patients’ details were anonymized and kept confidential. Prior to the start of study, an exemption of written informed consent for the use of tissue samples was obtained from the Institutional Review and Ethics Committee, Faculty of Medicine, University of Mutah. This study was performed in compliance with the guidelines of the Declaration of Helsinki (2013).

### Immunohistochemistry

Sections were deparaffinized and rehydrated by consecutive incubations in xylene with serial concentrations of ethanol. The samples were immersed in 3% hydrogen peroxide for 5 minutes to block the endogenous peroxidase activity. The tissues underwent antigen retrieval by being microwaved at 600 W for 20 minutes in a citrate buffer (10 mM citrate buffer, pH 6). Following a wash in phosphate-buffered saline, the sections were treated with normal blocking serum (1.5%) for 20 minutes at room temperature. After that, rabbit polyclonal antibodies specific for CYP1B1 (NBP1-85496, Novus Biological) were applied to each section at a concentration of 10 μg/mL for 1 hour at room temperature. After washing in phosphate-buffered saline, the sections were covered with ImmPRESS (peroxidase) polymer goat antirabbit IgG reagent for 30 minutes (MP-7451, Vector Laboratories). To visualize immunoreactivity, the sections were treated with 3,39-diaminobenzidine tetrahydrochloride chromogen solution for 2-5 minutes. The sections were then counterstained with Harris’s hematoxylin solution and mounted with coverslips. Negative control samples were designed by incubating sections with normal serum instead of the primary antibody to analyze the binding of the secondary antibody.

### Inhibition of CYP1B1 Immunoreactivity

To confirm specific binding of the primary antibody used, CYP1B1 antibody was preincubated with the human CYP1B1 blocking peptide (NBP1-85496PEP). The resulting antibody-peptide mix was then applied in place of CYP1B1 antibody to prevent subsequent primary antibody binding to CYP1B1 epitope in sample. The strength of staining was compared between the samples stained with CYP1B1 antibody and blocked antibody.

### Scoring

Immunohistochemical staining was semiquantitatively evaluated by 3 independent pathologists. Cells displaying cytoplasmic immunoreactivity were considered positive for CYP1B1 expression. The density and the degree of CYP1B1 expression in the tissue specimens were scored using the following scale: negative (0), low (1), moderate (2), and high (3). Tissue sections showing negative or less than 5% expression were assigned a score of 0 (negative). A score of 1 (low) was applied to tissue sections showing 5%-33% CYP1B1 expression. A score of 2 (moderate) indicated that tissue sections had 34%-66% CYP1B1 expression. Tissue sections displaying more than 67% CYP1B1 expression were assigned a score of 3 (high).

### Statistical Analysis

SPSS version 16 (SPSS Inc) was used for data analysis. Multiple variables were represented in simple measures of frequency and percentage. When applicable, Pearson chi-square test and one-way analysis of variance test were used to measure differences between the multiple variables, and *P* values less than .05 were considered statistically significant.

## Results

### Baseline Demographic and Clinicopathologic Features

Data on demographic and clinicopathologic features were available for 110 patient cases. These cases included 100 patients diagnosed with cervical cancer and 10 cases with normal cervix pathology presented as control. These normal cervical tissues comprised 2 (20%) cases of normal cervical tissues adjacent to tumors, and the remaining 8 (80%) were normal healthy cervical tissues. The average age of the patients was 50 (SD 10.2) years (Table 1). Approximately 62.7% (69/110) of the patients were younger than 50 years, while 37.3% (41/110) of the patients were older than 50 years. The most frequent subtype of cervical cancer among patients in this study was squamous cell carcinoma (95/110, 86.4%). Other subtypes included 1.8% (2/110) endometrioid adenocarcinomas and 2.7% (3/110) adenocarcinomas. Most of the cervical cancer cases were of histological grade III (64/100, 64.0%) and grade II (27/100, 27.0%), whereas grade I comprised only 9.0% (9/100) of the cases. Regarding the histological stage, a little more than half of the cases were at tumor stage I (56/100, 56.0%), whereas 38.0% (38/100) and 6.0% (6/100 cases) of the cases were at stage II and stage III, respectively. Moreover, 59.0% (59/100) of patients had tumors confined to the uterus (T1). The remaining cases were distributed as follows: 38.0% (38/100) of the patients had tumors that invaded beyond the uterus but not to the pelvic wall or vagina (T2) and 3.0% (3/100) of the patients had tumors that invaded the pelvic wall or vagina (T3). To facilitate statistical comparison, the status of lymph node metastasis was either classified as lymph node–positive (95/100, 95.0%) or lymph node–negative (5/100, 5.0%). The pathology evaluation demonstrated that all patients enrolled in this study were negative for distant metastasis.

**Table 1:** Baseline demographic and clinicopathologic features of patients in this study.

Patient characteristics		Negative CYP1B1^a^ expression, n (%)	Low CYP1B1 expression, n (%)	Moderate CYP1B1 expression, n (%)	High CYP1B1 expression, n (%)	*P* value	
**Age (years)**							.70
	<50 (n=69)	8 (12)	11 (16)	12 (17)	38 (55)		
	≥50 (n=41)	9 (22)	9 (22)	6 (15)	17 (41)		
**Pathology subtype**							.01
	Squamous cell carcinoma (n=95)	9 (10)	18 (19)	18 (19)	50 (53)		
	Adenocarcinoma (n=3)	0 (0)	0 (0)	0 (0)	3 (100)		
	Endometrioid adenocarcinoma (n=2)	0 (0)	0 (0)	0 (0)	2 (100)		
	Normal (n=10)	8 (80)	2 (20)	0 (0)	0 (0)		
**Histological grade**							.03
	I (n=9)	1 (6)	0 (0)	1 (6)	7 (13)		
	II (n=27)	3 (18)	9 (45)	4 (22)	11 (20)		
	III (n=64)	5 (29)	9 (45)	13 (72)	37 (67)		
**Histological stage**							.01
	I (n=56)	3 (17)	10 (50)	11 (61)	32 (58)		
	II (n=38)	6 (35)	5 (25)	6 (33)	21 (38)		
	III (n=6)	0 (0)	3 (15)	1 (6)	2 (4)		
**Tumor node metastasis histological stage**							.03
	T1 (n=59)	3 (18)	11 (55)	12 (67)	33 (60)		
	T2 (n=38)	6 (35)	5 (25)	6 (33)	21 (38)		
	T3 (n=3)	0 (0)	2 (10)	0 (0)	1 (2)		
**Lymph node metastasis**							.01
	Negative (n=95)	9 (53)	5 (75)	31 (33)	54 (98)		
	Positive (n=5)	0 (0)	3 (15)	1 (6)	1 (2)		
**Distant metastasis**							.13
	Negative (n=100)	9 (100)	18 (100)	18 (100)	55 (100)		
	Positive (n=0)	0 (0)	0 (0)	0 (0)	0 (0)		
**Human papilloma virus 16/18 status**							.04
	Low (n=32)	10 (31)	3 (9)	4 (13)	15 (47)		
	Medium (n=46)	3 (7)	7 (15)	7 (15)	29 (63)		
	High (n=32)	1 (3)	3 (9)	5 (16)	23 (72)		
**Ki67 status**							.75
	Low (n=56)	12 (71)	12 (60)	8 (44)	24 (44)		
	Moderate (n=36)	5 (29)	5 (25)	7 (39)	19 (35)		
	High (n=18)	0 (0)	3 (15)	3 (17)	12 (22)		

^a^CYP1B1: cytochrome P450 1B1.

### CYP1B1 Expression and Its Relation to Demographic and Clinicopathologic Features

**Figure 1:**
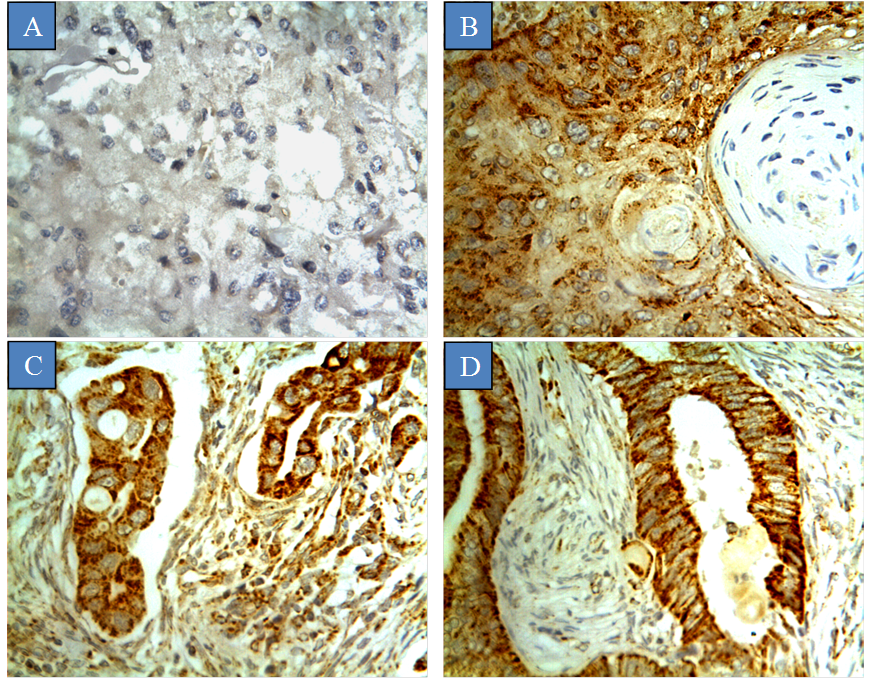
Cytochrome P450 1B1 expression in different types of cervical cancer. Tumors were classified on the basis of the histological subtype. (A) Normal cervix tissue, (B) squamous cell carcinoma, (C) adenocarcinoma, and (D) endometrioid adenocarcinoma.

**Figure 2:**
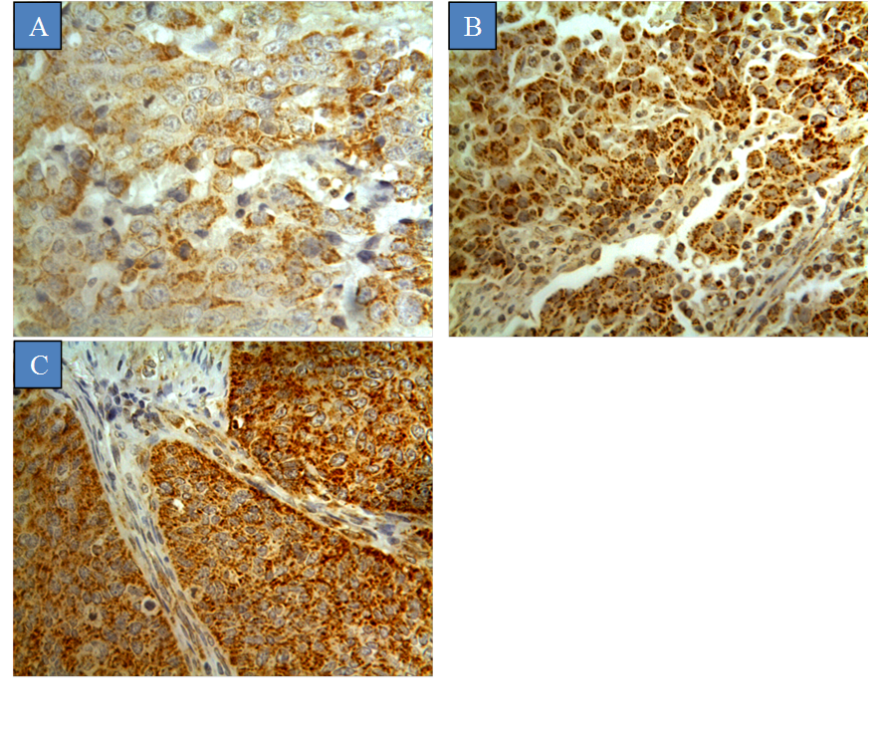
Cytochrome P450 1B1 expression in different grades of cervical cancer. Tumors were classified on the basis of histological grades. (A) Grade I, (B) grade II, and (C) grade III.

CYP1B1 expression was mainly localized to the membrane or cytoplasm, with no significant immunoreactivity observed in the nucleus (Figure 1). Staining was characterized as intense, and no evidence of heterogeneity was observed across all sections examined. The expression of CYP1B1 was verified by inhibiting the immunoreactivity with CYP1B1 protein as a blocking step (Multimedia Appendix 1). CYP1B1 was identified in 91.0% (91/100) of the patient samples, while others had no expression (9/100, 9.0%). Half of the cases showed high CYP1B1 expression (55/100, 55.0%), while the remaining cases exhibited moderate (18/100, 18.0%) and low (18/100, 18.0%) expression. Normal cervical tissues displayed low CYP1B1 expression in 20% of the cases (2/10). Interestingly, these CYP1B1-positive normal tissues were normal cervical tissues adjacent to tumors, while normal healthy cervical tissues displayed no CYP1B1 expression at all. The association between CYP1B1 expression in cervical cancer and different clinicopathological characteristics is shown in Table 1. There were significant associations between CYP1B1 expression and tumor pathological subtype (*P*=.01), tumor grade (*P*=.03), lymph node metastasis (*P*=.01), and HPV 16/18 expression (*P*=.04). CYP1B1 expression in normal cervical tissues and all subtypes of cervical cancer tissues was significantly different (*P*=.01). CYP1B1 expression was detected in 91% (86/95) of the squamous cell carcinomas and all cases of adenocarcinomas and endometrioid adenocarcinomas. Moreover, CYP1B1 was highly expressed in grade III (59/100, 59.0%) adenocarcinomas compared to grade II (24/100, 24.0%) adenocarcinomas and grade I adenocarcinomas (8/100, 8.0%) (Figure 2). All patients having tumors with metastasis to the lymph node exhibited a high rate of CYP1B1 expression. Moreover, there was a high frequency of CYP1B1 expression in cases expressing moderate (29/46, 63%) to high (23/32, 72%) incidence of HPV 16/18. In addition, no significant associations were found between CYP1B1 expression and age of patients, tumor stage, tumor depth of invasion, and status of Ki67.

## Discussion

Cervical cancer remains the fourth most frequent cause of cancer mortality in females worldwide, thereby representing the most prevalent malignancy in adult women in low-income countries [1,3]. The morbidity and mortality due to cervical cancer are rising globally, particularly in low-income countries owing to the lack of or improper screening and vaccination programs as well as absence of inadequate treatment standard guidelines [4,5]. In this regard, the focus of current cancer research is the discovery of novel therapies for cervical cancer based on the alterations in the molecular biology of the cancer cells. Of interest is the emerging research about the potential facets of aberrant CYP1B1 expression in several cancers and its promising role in anticancer drug development [11,34]. The main finding of this study is that CYP1B1 was selectively expressed in cervical cancers and not in normal cervical tissues, thereby making it a potential target for future development of CYP1B1-based therapeutic strategies.

Several studies have indicated selective CYP1B1 expression in many cancers [11,12,14,16,19-21]. However, neither of these studies have investigated CYP1B1 expression in cervical cancer. In this study, for the first time, we describe high CYP1B1 expression in cervical cancers, in particular, in squamous cell carcinoma. This pathohistological subtype is the most frequent cervical cancer and constitutes 90% of all cancers found at this site [35]. Other cervical cancer subtypes included in this study also displayed CYP1B1 overexpression. Importantly, low CYP1B1 expression was exhibited in normal cervical tissues; this particularly applied to normal cervical tissues adjacent to tumors. Conversely, no expression at all was found in normal healthy cervical tissues (non–tumor-bearing tissues). A similar oncoantigen expression in normal tissues adjacent to tumors and negative CYP1B1 expression in normal healthy tissues has been reported in many studies [36,37]. Taken together, normal and tumor cervical tissues showed a significant difference in CYP1B1 expression (*P*=.01). A similar significant difference has also been reported in other studies [18-21]. In light of all these reports, differences in the CYP1B1 expression between normal and tumor cervical tissues supports the development of potential therapeutic approaches for cervical cancer.

Abundant evidence shows that CYP1B1 expression is associated with aggressive behavior and poor survival in several cancers [38,39]. In our study, significant associations were determined between CYP1B1 expression and tumor grade, lymph node metastasis, and HPV 16/18 expression. A higher rate of CYP1B1 expression was observed in advanced grades of disease than that in the early grades of disease. Other studies have also reported such an observation, thereby demonstrating a high frequency of CYP1B1 expression in patients with advanced grades of non–small cell lung cancer and renal cancer [22,23]. Moreover, CYP1B1 expression was found to be high in all patients with lymph node metastasis. Another interesting finding was that CYP1B1 was highly expressed in patients with a high prevalence of HPV 16/18. These results suggest that the CYP1B1 enzyme may have a role in cervical cancer development.

The fact that CYP1B1 is overexpressed in many types of cancers but is either found in at minute levels or absent in normal tissues makes it a promising therapeutic target [40]. This applies particularly to CYP1B1, where it metabolizes many xenobiotics to procarcinogens, leading to the development of many cancers [41]. Moreover, CYP1B1 is responsible for the metabolic inactivation of many chemotherapeutic agents, including paclitaxel, flutamide, docetaxel, mitoxantrone, and tamoxifen, consequently mediating drug resistance [40,41]. Therefore, the development of CYP1B1-based therapeutic strategies would be a great advantage for the treatment of cancers expressing CYP1B1. These therapeutic strategies are CYP1B1 inhibitors, CYP1B1-based immunotherapy, and CYP1B1-directed prodrugs [40,41]. The application of CYP1B1 inhibitors to overcome anticancer drug resistance has shown promising results in both in vitro and in vivo studies. In this regard, tetramethoxystilbene has been shown to decrease the growth of xenografted tamoxifen-resistant MCF-7 cells by 53% [42]. Moreover, in vitro and in a xenograft model, α-naphthoflavone suppressed paclitaxel resistance and promoted the sensitivity of ovarian cells to paclitaxel [43]. Docetaxel resistance in MCF-7/1B1 cells was abolished by a water-soluble analog of α-naphthoflavone, which was mediated by increased CYP1B1 expression [40,44]. Importantly, CYP1B1 is also a target for immunotherapy for late stages of cancer, where CYP1B1 vaccination increases the patients’ overall outcome. This CYP1B1-based vaccine (ZYC300) activated the immune system, producing cytotoxic T lymphocytes against cancer cells expressing CYP1B1 [40]. Another, probably more ideal, strategy for CYP1B1-directed anticancer therapy is to rationally design prodrugs selectively activated by CYP1B1 in tumor tissues to cytotoxic metabolites without affecting normal cells [40]. Several attempts were successful in identifying potential CYP1B1-mediated prodrugs such as DMU-135 and resveratrol [45,46]. However, these prodrugs are still under preclinical investigation. Taken together, the current knowledge and understanding of cancer-selective CYP1B1 expression may hopefully lead to the discovery of novel CYP1B1-based therapeutic strategies.

In conclusion, this study is the first to characterize CYP1B1 overexpression in cervical cancers compared to low CYP1B1 expression in the corresponding normal cervical tissues adjacent to tumors. Normal healthy cervical tissues displayed negative CYP1B1 expression. Moreover, this unique CYP1B1 expression was frequently found in advanced grades of disease and in patients having metastasis to the lymph node. Interestingly, a high rate of CYP1B1 expression was observed in patients with high HPV 16/18 prevalence, which is a novel finding. Importantly, strong CYP1B1 differential expression between normal and tumor cervical tissues makes it a potential therapeutic target for the development of novel CYP1B1-based therapeutic strategies.

## Appendix

Multimedia Appendix 1 Validation of cytochrome P450 1B1 antibody specificity for immunohistochemical analysis. Immunohistochemical staining in the absence (A) and presence (B) of cytochrome P450 1B1 protein. Immunohistochemical staining was abolished in the presence of the cytochrome P450 1B1 protein.

## Abbreviations

CYP1B1: cytochrome P450 1B1
HPV: human papilloma virus
