# Supplementary figures and images for: Cytochrome P450 1B1 Overexpression in Cervical Cancers: Cross-sectional Study
Source: Interact J Med Res. 2021 Oct 12;10(4):e31150. doi: 10.2196/31150 (PMC8548976; doi:10.2196/31150)

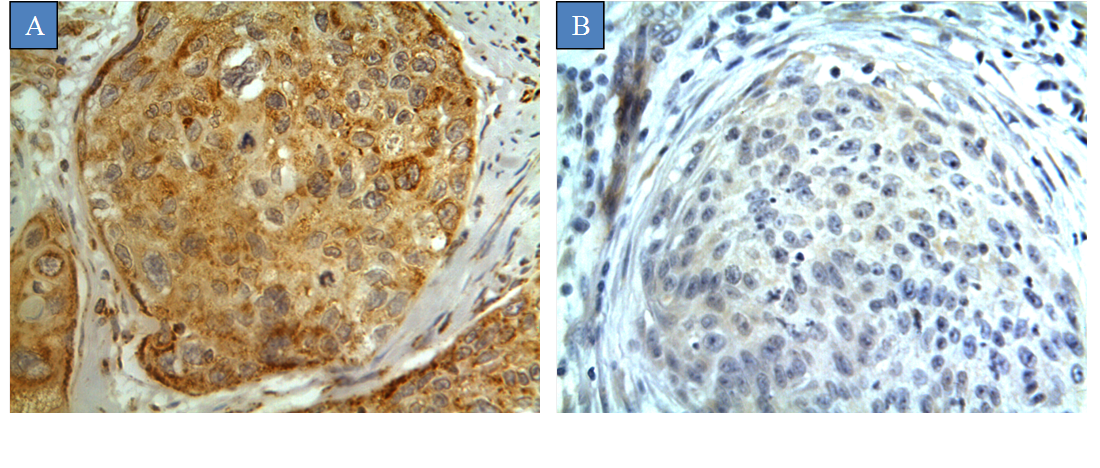

Supplement: Multimedia Appendix 1 [file ijmr_v10i4e31150_app1.png]
